# Abdominal CT-derived metabolic phenotypes are associated with the MRI-derived brain age gap in women

**DOI:** 10.3389/fnagi.2026.1893253

**Published:** 2026-08-04

**Authors:** Chongwon Pae, Minchul Kim, Inpyeong Hwang, Joon Ho Moon, Kyu Sung Choi

**Affiliations:** 1 Department of Radiology, Seoul National University Hospital, Seoul, Republic of Korea; 2 Brain Imaging Center, Seoul National University, Seoul, Republic of Korea; 3 Department of Radiology, Kangbuk Samsung Hospital, Sungkyunkwan University School of Medicine, Seoul, Republic of Korea; 4 Department of Radiology, Seoul National University College of Medicine, Seoul, Republic of Korea; 5 Department of Endocrinology and Metabolism, Seoul National University Bundang Hospital, Seongnam, Republic of Korea; 6 Department of Internal Medicine, Seoul National University College of Medicine, Seoul, Republic of Korea; 7 Healthcare AI Research Institute, Seoul National University Hospital, Seoul, Republic of Korea

**Keywords:** abdominal adiposity, body composition, brain age gap, computed tomography, liver-to-spleen attenuation ratio

## Abstract

**Background:**

Metabolic dysfunction is increasingly recognized as a systemic contributor to adverse brain aging; however, conventional clinical markers may not fully capture tissue-level variations in liver attenuation, abdominal adiposity, and muscle quality. We examined whether abdominal CT-derived metabolic phenotypes were associated with MRI-derived brain age gap (BAG) in women.

**Methods:**

This retrospective health-screening cohort included 1,280 women who underwent both abdominal CT and brain MRI between 2019 and 2024. Predicted brain age was estimated using brainageR, and the BAG was calculated as predicted brain age minus chronological age. CT-derived phenotypes were extracted from dual-energy CT virtual non-contrast images using DeepCatch software. The primary CT phenotypes were FatRatio, defined as the abdominal fat area relative to the fat plus skeletal muscle area, and liver-to-spleen attenuation ratio.

**Results:**

In models adjusted for age, parity, body mass index, hemoglobin A1c, triglycerides, high-density lipoprotein (HDL) cholesterol, smoking, and alcohol use, a higher FatRatio was associated with a larger BAG (*β* = 0.108, *p* = 0.007), whereas a higher liver-to-spleen attenuation ratio was associated with a lower BAG (*β* = −0.123, *p* < 0.001). In joint models including both primary CT phenotypes and the same clinical covariates, FatRatio (*β* = 0.095, *p* = 0.037) and liver-to-spleen attenuation ratio (*β* = −0.115, *p* = 0.001) retained independent associations. These findings persisted after age-bias correction of BAG and exclusion of extreme BAG outliers. CT-derived markers added modest but significant explanatory value beyond conventional clinical covariates.

**Conclusion:**

These findings suggest that abdominal CT-derived liver attenuation and relative adiposity phenotypes may capture systemic tissue-level metabolic variations relevant to MRI-derived brain aging in women.

## Introduction

Metabolic dysfunction is increasingly recognized as a systemic contributor to adverse brain aging. Brain-age studies have linked metabolic dysfunction–associated steatotic liver disease (MASLD), hepatic steatosis-related traits, and adipose tissue distribution with older-appearing brain phenotypes (Beck et al., 2022; Moran et al., 2024; Wang et al., 2025). Liver-focused studies have also associated MASLD or subclinical liver traits with cognitive, structural, or hemodynamic brain markers (George et al., 2022; Yilmaz et al., 2023; Mikkelsen et al., 2025). However, MASLD is a clinical-metabolic disease construct rather than a CT attenuation measure alone, and imaging-derived liver attenuation phenotypes should be distinguished from formal MASLD diagnoses. Visceral adiposity and obesity have been associated with lower brain volume and altered white matter microstructure (Debette et al., 2010; Dekkers et al., 2019). However, commonly used clinical indicators, such as body mass index (BMI) and blood biomarkers, may not fully capture tissue-level heterogeneity in fat distribution, liver attenuation, and muscle quality.

Routine abdominal CT offers an opportunity to extract quantitative metabolic information from imaging studies already performed for clinical or health-screening purposes. Value-added opportunistic CT screening has been proposed for extracting additional risk information from routine scans (Pickhardt et al., 2021b, 2023; Pickhardt, 2022). Automated body-composition methods can quantify visceral and subcutaneous fat, muscle attenuation, and liver attenuation at scale (Lee et al., 2021; Magudia et al., 2021; Pickhardt et al., 2021a). CT-derived visceral adiposity and hepatic steatosis phenotypes are associated with metabolic syndrome and cardiometabolic outcomes (Pickhardt et al., 2012; Pickhardt et al., 2020). Muscle attenuation is central to CT-based sarcopenia and muscle quality (Boutin and Lenchik, 2020). These CT-derived phenotypes may therefore provide complementary information beyond anthropometric and laboratory measures. When paired with brain MRI, these CT-derived phenotypes may provide a systemic metabolic context for interpreting interindividual variations in brain aging.

Brain age gap (BAG), defined as predicted brain age minus chronological age, summarizes whether an individual’s brain appears older or younger than expected for chronological age. Brain-predicted age has been used as a population-level neuroimaging phenotype associated with mortality, dementia risk factors, cardiometabolic burden, and disease-related vulnerability (Cole et al., 2017, 2018; Stefaniak et al., 2024). This broader context of metabolic brain aging is clinically relevant because dementia-prevention frameworks increasingly emphasize modifiable cardiometabolic risk factors throughout life (Livingston et al., 2024). Methodological reviews of Brain Age Gap Estimation (BrainAGE) have highlighted both the clinical relevance of brain-predicted age and the need for careful interpretation of model-derived age gaps (Franke and Gaser, 2019). Because BAG may be sensitive to model calibration and age-related prediction bias, studies using BAG should explicitly address age-bias correction and calibration (Beheshti et al., 2019; Smith et al., 2019; de Lange and Cole, 2020). Public brain-age tools may also differ in accuracy and reliability, supporting the need for calibration diagnostics when applying them to independent cohorts (de Lange et al., 2022; Dorfel et al., 2023).

Despite growing interest in both opportunistic CT phenotyping and brain-age modeling, it remains unclear whether abdominal CT-derived metabolic phenotypes are associated with MRI-derived BAG. This question is particularly relevant in health-screening cohorts in which abdominal CT and brain MRI may be performed in the same individuals. Liver attenuation and adiposity phenotypes are especially relevant because they reflect complementary aspects of the systemic metabolic burden, including hepatic steatosis-related tissue attenuation and adverse fat distribution. Women-specific life-course factors may further shape later-life metabolic phenotypes, as pregnancy and childbirth have been associated with long-term changes in body composition and metabolic risk (Kim et al., 2016; Schindler et al., 2022). Prior findings relating parity to cognition, dementia, and brain age are heterogeneous (Bae et al., 2020; Ning et al., 2020). Accordingly, parity may be better treated as a female-specific life-course context than as a simple causal exposure for brain aging.

In this retrospective health-screening cohort of women, we examined whether abdominal CT-derived metabolic phenotypes, reflecting liver attenuation, abdominal adiposity, and muscle quality, were associated with MRI-derived BAG. We focused on the liver-to-spleen attenuation ratio and FatRatio as primary CT-derived metabolic phenotypes, tested whether these markers provided information beyond conventional clinical covariates, and evaluated an integrated CT burden score as a summary of adverse systemic metabolic phenotypes. We additionally examined parity as a female-specific life-course context rather than as a primary causal determinant of BAG.

## Materials and methods

### Study design and participants

This retrospective study included women who voluntarily underwent an institutional comprehensive health-screening program between January 2019 and December 2024. Participants were eligible if they had completed a health-screening package that included abdominal CT and brain MRI, and if abdominal CT images suitable for body-composition analysis and T1-weighted brain MRI scans suitable for *brainageR*-based brain-age estimation were available. The study was approved by the Institutional Review Board (IRB) of Seoul National University Hospital (No. 2504–076-1629), and the requirement for informed consent was waived because de-identified retrospective data were analyzed. This study was conducted in accordance with the principles of the Declaration of Helsinki.

Participants were excluded for prior structural brain pathology, focal brain lesions, MRI quality-control failure due to motion artifact, or missing body composition data. The primary female cohort included 1,280 women. BAG was available in 1,278 women, and valid parity information was available in 1,231 women.

### MRI brain age estimation

Brain MRI included T1-weighted structural imaging acquired under a uniform institutional health-screening MRI protocol. Brain MRI was performed using a GE Discovery MR750w 3.0 T system (GE HealthCare, Chicago, IL, USA) equipped with a 24-channel head coil. T1-weighted images were acquired using a three-dimensional fast spoiled gradient-echo sequence with the following parameters: repetition time 8.0 ms; echo time, 3.0 ms, inversion time, 450 ms; flip angle, 12°; field of view, 256 × 256 mm; acquisition matrix, 256 × 256; receiver bandwidth, 139.4 Hz/pixel; number of excitations, 1; sagittal slices, 154 to 172 depending on head size; slice thickness, 1 mm; and isotropic voxel size, 1 mm^3^.

Predicted brain age was estimated using *brainageR* version 2.1 (https://github.com/james-cole/brainageR), an open-access software package that generates brain age predictions from raw T1-weighted MRI scans (Cole et al., 2017; Cole et al., 2018). *BrainageR* consists of preprocessing and prediction stages. In the preprocessing stage, native T1-weighted images were segmented and spatially normalized using SPM12 (Wellcome Centre for Human Neuroimaging, UCL Queen Square Institute of Neurology, London, UK; https://www.fil.ion.ucl.ac.uk/spm/software/spm12/). For quality control, two-dimensional slice images of the segmentation and normalization outputs were generated using the FMRIB Software Library (FSL) *slicesdir* function. The normalized gray matter, white matter, and cerebrospinal fluid probability maps were loaded into *R* and converted into vectors. These vectors were masked using a 0.3 threshold from the mean image template on the *brainageR* training dataset and then combined.

In the prediction stage, the pretrained *brainageR* model was applied to the vectorized and masked images to estimate the predicted brain age for each participant. The *brainageR* model was trained on normalized brain volumetric maps from 3,377 healthy individuals across seven publicly available datasets using Gaussian process regression. Principal component analysis was used in the original model to retain components explaining 80% of the variance in brain volumes, resulting in 435 principal components. The corresponding rotation matrix was applied to the present imaging data to generate the predicted brain age. Further details regarding model development and validation are provided in the original *brainageR* studies and software documentation (Cole et al., 2017; Cole et al., 2018; Cole, 2019).

Brain age gap (BAG) was calculated as predicted brain age minus chronological age. Positive BAG values indicated an older-appearing brain relative to chronological age, whereas negative values indicated a younger-appearing brain. Because regression-based brain-age models can show age-related prediction bias, we evaluated quadratic age-bias-corrected BAG as a sensitivity outcome (Beheshti et al., 2019; Smith et al., 2019; de Lange and Cole, 2020). Quadratic age-bias-corrected BAG was calculated as the residual from the regression of raw BAG on chronological age and chronological age squared. In the primary regression models, BAG was standardized to allow comparisons across CT-derived predictors. Selected models were also repeated using unstandardized BAG in years to report the BAG-year effect sizes.

### Abdominal CT acquisition and CT-derived metabolic phenotypes

Abdominal CT was performed using a dual-source dual-energy CT scanner (SOMATOM Force; Siemens Healthineers, Erlangen, Germany) in the institutional health-screening setting. After intravenous administration of iobitridol (Xenetix 350; Guerbet) at 520 mg/kg body weight, followed by a saline flush, images were acquired during the single portal venous phase 70 s after contrast medium delivery. Data were acquired in dual-energy mode using 80 and 150 kVp settings. Virtual non-contrast images were generated from the dual-energy data using a dedicated Syngo.via post-processing system (Siemens Healthineers) and reconstructed with a 2-mm slice thickness. All CT-derived body composition and organ attenuation phenotypes used in this study were extracted from virtual non-contrast images. The same scanner model and standardized CT acquisition and reconstruction protocols were used throughout the study period.

**Figure 1:**
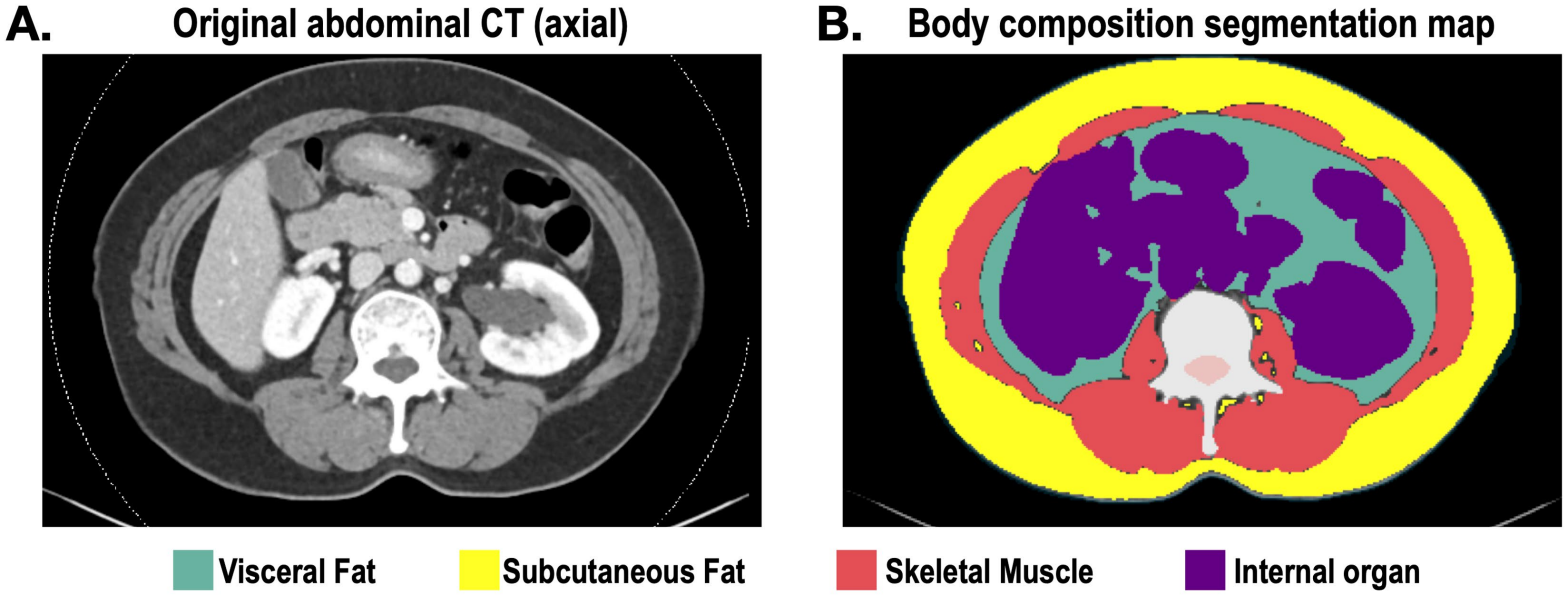
Representative abdominal CT image and automated body-composition segmentation. **(A)** Representative axial abdominal CT image. **(B)** Corresponding automated segmentation map illustrating the abdominal body-composition compartments used in this study, including visceral fat, subcutaneous fat, skeletal muscle, and internal organs. FatRatio was calculated as the sum of visceral and subcutaneous fat area divided by the sum of visceral fat, subcutaneous fat, and skeletal muscle area. AVFRatio was calculated as visceral fat area divided by the sum of visceral and subcutaneous fat area. Muscle attenuation was derived from the segmented skeletal muscle compartment. Liver-to-spleen attenuation ratio was calculated as the mean liver attenuation divided by the mean spleen attenuation using automatically segmented organ masks on virtual non-contrast CT images.

CT-derived metabolic phenotypes were obtained using DeepCatch (version 1.2.0.0, MEDICALIP, Seoul, South Korea), a deep learning-based application for body composition and organ attenuation phenotyping (Lee et al., 2021). Liver and spleen attenuation values were extracted from automatically segmented organ masks, and the liver-to-spleen attenuation ratio was calculated as the mean liver attenuation divided by the mean spleen attenuation. No participants were excluded for focal liver/spleen lesions or segmentation failure. A representative abdominal CT image and an automated body-composition segmentation map are provided in Figure 1 to illustrate the CT-derived body-composition phenotyping approach.

CT-derived phenotypes were selected to represent the complementary domains of metabolic body composition, including relative abdominal adiposity and distribution, liver-related attenuation, and muscle quality. Adiposity phenotypes included FatRatio, Abdominal Visceral Fat Ratio (AVFRatio), and visceral fat volume. FatRatio was defined as the total abdominal fat area divided by the sum of abdominal fat and skeletal muscle areas, calculated as (abdominal visceral fat area + subcutaneous fat area)/(abdominal visceral fat area + subcutaneous fat area + skeletal muscle area). AVFRatio was defined as the abdominal visceral fat area divided by the total abdominal fat area on a single CT slice and calculated as the abdominal visceral fat area/(abdominal visceral fat area + subcutaneous fat area). Liver-related attenuation was assessed using the liver-to-spleen attenuation ratio, calculated as the mean liver attenuation divided by the mean spleen attenuation on virtual non-contrast images. Muscle quality was represented by muscle attenuation, a CT radiodensity phenotype related to intramuscular lipid infiltration and tissue quality (Goodpaster et al., 2000; Aubrey et al., 2014). Quantitative CT muscle assessment has also been reviewed as a broader imaging approach to muscle quality (Engelke et al., 2018). The muscle-to-visceral fat ratio was calculated as single-slice abdominal muscle volume divided by single-slice abdominal visceral fat volume and was evaluated as an exploratory secondary derived marker reflecting the relative balance between muscle and visceral fat compartments.

The primary CT-derived phenotypes were FatRatio and liver-to-spleen attenuation ratio. FatRatio was selected as the primary relative adiposity phenotype because it captures the proportion of abdominal fat area within the fat- and muscle-containing abdominal body composition area. AVFRatio, visceral fat volume, muscle attenuation, and muscle-to-visceral fat ratio were evaluated as secondary CT-derived markers. The muscle-to-visceral fat ratio was considered exploratory because it was a derived ratio rather than a directly measured CT phenotype. The liver-to-spleen attenuation ratio was interpreted as a spleen-normalized hepatic attenuation phenotype relevant to hepatic steatosis. Because MASLD requires a broader clinical-metabolic definition, including cardiometabolic risk context, consideration of alcohol intake, and alternative liver disease etiologies, the liver-to-spleen attenuation ratio was not treated as a definitive MASLD diagnosis (Eslam et al., 2020; Choi et al., 2021).

To further clarify the interpretation of the ratio-based adiposity phenotypes, secondary abdominal fat compartment decomposition analyses were performed. These analyses evaluated visceral fat volume, subcutaneous fat volume, total abdominal fat volume, skeletal muscle volume, FatRatio, AVFRatio, and muscle attenuation. Because abdominal fat volume variables were right-skewed, visceral fat volume, subcutaneous fat volume, and total abdominal fat volume were log-transformed before standardization. These analyses were interpreted as secondary decomposition analyses to assess whether the FatRatio-BAG association could be explained by isolated visceral fat, subcutaneous fat, total abdominal fat, or relative fat predominance over muscle-containing tissue compartments.

Exploratory adipose tissue attenuation analyses were performed to evaluate CT-derived adipose tissue quality beyond the adipose tissue quantity. Mean subcutaneous fat attenuation and visceral fat attenuation values were derived from the corresponding segmented adipose tissue compartments on virtual non-contrast CT images. These attenuation measures were evaluated using the same age/parity-adjusted and clinically adjusted model structures as in the primary analyses. Quantity-adjusted models additionally included the corresponding log-transformed compartment volumes. Additional exploratory models were further adjusted for FatRatio and liver-to-spleen attenuation ratio. The correlation between subcutaneous fat attenuation and visceral fat attenuation was examined to assess the overlap between the two attenuation measures.

### Integrated CT burden score

An exploratory integrated CT burden score was constructed for clinical interpretability rather than as a primary mechanistic or predictive model. One point was assigned for each adverse CT phenotype: high FatRatio, low liver-to-spleen attenuation ratio, and low muscle attenuation, using sample quartile-based thresholds. These three components were selected to summarize the complementary CT-derived domains of adverse metabolic body composition, including relative abdominal adiposity, hepatic attenuation, and muscle quality. The total score ranged from 0 to 3, with higher scores indicating a more adverse CT-derived metabolic phenotype. Trend analyses modeled the CT burden score as an ordinal variable ranging from 0 to 3. A secondary categorical contrast compared women with score 0 versus score 2 or greater, excluding a score of 1, to separate the low and high CT burden groups.

### Clinical covariates and reproductive history

Clinical covariates included chronological age, parity category, BMI, hemoglobin A1c, triglycerides, HDL cholesterol, smoking status, and alcohol use. These variables were selected to account for conventional adiposity and metabolic risk factors while testing whether CT-derived phenotypes provided additional tissue-level information.

Reproductive history was obtained using standardized self-reported health-screening questionnaires. Parity was defined as the self-reported number of live births and was categorized as 0, 1, 2, 3, or more births. Parity was treated as a female-specific life-course context and an adjustment/context variable, not as the primary causal exposure. Direct parity-BAG associations were transparently reported, including null findings.

Models used complete-case analysis for the variables included in each model. Clinically adjusted models required non-missing BAG, the relevant CT predictor, age, parity category, BMI, hemoglobin A1c, triglycerides, HDL cholesterol, smoking status, and alcohol use.

### Statistical analysis

The primary analysis tested whether the two primary CT-derived phenotypes, FatRatio and liver-to-spleen attenuation ratio, were associated with raw BAG. Continuous CT predictors and BAG were standardized before regression analyses, unless BAG-year units were reported.

Baseline characteristics were summarized as mean ± standard deviation, median [interquartile range], or number and percentage, as appropriate. For group comparisons, normally distributed continuous variables were compared using one-way analysis of variance, non-normally distributed continuous variables were compared using the Kruskal-Wallis test, and categorical variables were compared using the chi-square test or Fisher’s exact test when appropriate.

Linear regression models followed two adjustment levels. Model 1 was adjusted for chronological age and parity category. Model 2, the primary clinically adjusted model, additionally included BMI, hemoglobin A1c, triglycerides, high-density lipoprotein (HDL) cholesterol, smoking status, and alcohol use. Joint CT models included FatRatio, liver-to-spleen attenuation ratio, and muscle attenuation simultaneously with clinical covariates to evaluate whether individual CT phenotypes provided independent information. Regression coefficients were tested using Wald *t*-tests, and variance inflation factors (VIFs) were calculated to assess multicollinearity.

AVFRatio, visceral fat volume, subcutaneous fat volume, total abdominal fat volume, skeletal muscle volume, muscle attenuation, and muscle-to-visceral fat ratio were analyzed as secondary CT-derived markers. Subcutaneous fat attenuation and visceral fat attenuation were additionally evaluated as exploratory CT-derived adipose tissue attenuation markers to assess adipose tissue quality. The CT burden score, high-BAG logistic models, and parity interaction analyses were considered exploratory.

Linear-model diagnostics were reviewed using residual distributions, quantile-quantile plots, and residual-versus-fitted plots. Unless otherwise specified, two-sided *p*-values less than 0.05 were considered statistically significant.

Incremental explanatory value was assessed by comparing a base clinical model with CT-augmented models using adjusted *R*^2^, *Δ* adjusted *R*^2^, partial *R*^2^, and nested model comparison *F*-tests. Selected models were repeated using unstandardized BAG in years to estimate the expected BAG-year difference associated with a 1 SD higher CT-derived phenotype or a 1-point higher CT burden score.

Robustness analyses were used to evaluate whether the primary CT-BAG associations were sensitive to brain-age calibration, extreme BAG values, and the temporal interval between abdominal CT and brain MRI. First, the primary models were repeated using quadratic age-bias-corrected BAG as the outcome variable. Second, the primary models were repeated after excluding extreme BAG outliers, defined as absolute BAG z-scores greater than 3. Third, the primary clinically adjusted models were repeated after additional adjustments for the absolute CT-MRI interval. As an additional sensitivity analysis, the primary models were repeated in participants who underwent abdominal CT and brain MRI on the same day. As an additional sensitivity analysis addressing glucose-metabolism covariate selection, the primary clinically adjusted models were repeated after replacing hemoglobin A1c with fasting glucose.

### Exploratory and contextual analyses

Exploratory high-BAG analyses defined high BAG as the top quartile of BAG. Logistic regression models estimated the odds ratios for high BAG according to FatRatio, liver-to-spleen attenuation ratio, and CT burden score. *p*-values for logistic regression odds ratios were based on Wald tests. Apparent area under the curve (AUC) values were calculated for exploratory discrimination only and were not interpreted as clinical prediction performance.

Contextual parity analyses included direct parity-BAG and age-by-parity interaction models. Direct parity-BAG associations were evaluated in the full parity-available sample and only in parous women. Age-by-parity interaction models were used to assess whether parity was related to age-related patterning of BAG rather than to mean BAG differences. In the BAG interaction models, AVFRatio was included as an additional covariate to account for abdominal fat distribution. Sensitivity analyses were performed in parous women only and in older age-restricted subgroups.

## Results

### Study population

A total of 1,280 women were included in the analysis cohort. BAG was available in 1,278 women, and valid parity information was available in 1,231 women. Baseline characteristics according to chronological age tertiles are summarized in Table 1. Across age tertiles, BMI, hemoglobin A1c, FatRatio, AVFRatio, visceral fat volume, HDL cholesterol, muscle attenuation, and parity category differed significantly. Mean predicted brain age was 50.93 years, mean chronological age was 61.22 years, and mean BAG was −10.29 years with an SD of 7.22 years. BAG differed across age tertiles in descriptive comparisons, whereas it showed no strong linear association with chronological age (*r* = 0.040, *p* = 0.157). Detailed cohort-level calibration diagnostics are provided in Supplementary Table S1 and Supplementary Figure S1.

**Table 1:** Baseline characteristics according to chronological age tertiles.

Characteristic	Overall	Age tertile 1 ≤ 58.00 years	Age tertile 2 >58.00 to ≤65.00 years	Age tertile 3 >65.00 years	*p*-value
Participants, *n*	1,280	439	463	378	–
Age, years	61.2 ± 8.6	52.2 ± 5.8	61.9 ± 2.0	70.9 ± 4.3	–
BMI, kg/m^2^	23.51 ± 3.42	23.07 ± 3.50	23.41 ± 3.41	24.15 ± 3.24	<0.001***
Hemoglobin A1c, %	5.70 [5.40, 6.00]	5.50 [5.30, 5.80]	5.70 [5.50, 6.10]	5.80 [5.50, 6.20]	<0.001***
Triglycerides, mg/dL	82 [62, 113]	81 [61, 106]	82 [61, 120]	82 [63, 114]	0.304
HDL cholesterol, mg/dL	58.9 ± 13.7	61.3 ± 14.6	58.7 ± 13.2	56.2 ± 12.8	<0.001***
Predicted brain age, years	50.9 ± 11.4	42.0 ± 8.9	50.4 ± 7.2	61.9 ± 8.5	<0.001***
BAG, years	−10.3 ± 7.2	−10.2 ± 7.6	−11.5 ± 6.9	−8.9 ± 6.9	<0.001***
FatRatio	0.70 [0.63, 0.75]	0.67 [0.60, 0.73]	0.70 [0.64, 0.74]	0.72 [0.66, 0.76]	<0.001***
AVFRatio	0.32 [0.23, 0.41]	0.27 [0.19, 0.36]	0.33 [0.25, 0.41]	0.37 [0.29, 0.45]	<0.001***
Visceral fat volume, cm^3^	2,084 [1,351, 3,032]	1,664 [1,027, 2,493]	2,197 [1,459, 2,997]	2,440 [1,742, 3,354]	<0.001***
Liver-to-spleen attenuation ratio	1.14 [1.03, 1.27]	1.12 [1.03, 1.24]	1.14 [1.02, 1.27]	1.17 [1.04, 1.30]	0.007**
Muscle attenuation, HU	36.0 ± 6.5	39.1 ± 5.1	36.0 ± 5.9	32.4 ± 6.8	<0.001***
Parity category, *n* (%)					<0.001***
0 births	100 (8.1%)	58 (13.7%)	30 (6.7%)	12 (3.3%)	
1 birth	121 (9.8%)	59 (14.0%)	42 (9.4%)	20 (5.6%)	
2 births	667 (54.2%)	244 (57.8%)	269 (59.9%)	154 (42.8%)	
≥3 births	343 (27.9%)	61 (14.5%)	108 (24.1%)	174 (48.3%)	
Smoking status, *n* (%)					0.017*
Never smoker	1,001 (94.8%)	352 (91.9%)	360 (95.5%)	289 (97.6%)	
Former smoker	30 (2.8%)	18 (4.7%)	9 (2.4%)	3 (1.0%)	
Current smoker	25 (2.4%)	13 (3.4%)	8 (2.1%)	4 (1.4%)	
Alcohol use status, *n* (%)					<0.001***
Never drinker	805 (76.1%)	261 (68.0%)	291 (77.0%)	253 (85.5%)	
Former drinker	11 (1.0%)	3 (0.8%)	4 (1.1%)	4 (1.4%)	
Current drinker	242 (22.9%)	120 (31.2%)	83 (22.0%)	39 (13.2%)	

BAG, brain age gap; HDL, high-density lipoprotein; HU, Hounsfield units; IQR, interquartile range.

Values are presented as mean ± SD, median [IQR], or *n* (%), as appropriate.

FatRatio was defined as the total abdominal fat area divided by the sum of abdominal fat and skeletal muscle areas: (abdominal visceral fat area + subcutaneous fat area)/(abdominal visceral fat area + subcutaneous fat area + skeletal muscle area).

AVFRatio was defined as the abdominal visceral fat area divided by the total abdominal fat area on a single CT slice: abdominal visceral fat area/(abdominal visceral fat area + subcutaneous fat area).

Bolded statistical significance is indicated as *p < 0.05, **p < 0.01, and ***p < 0.001.

### Primary CT-derived phenotype associations with BAG

**Figure 2:**
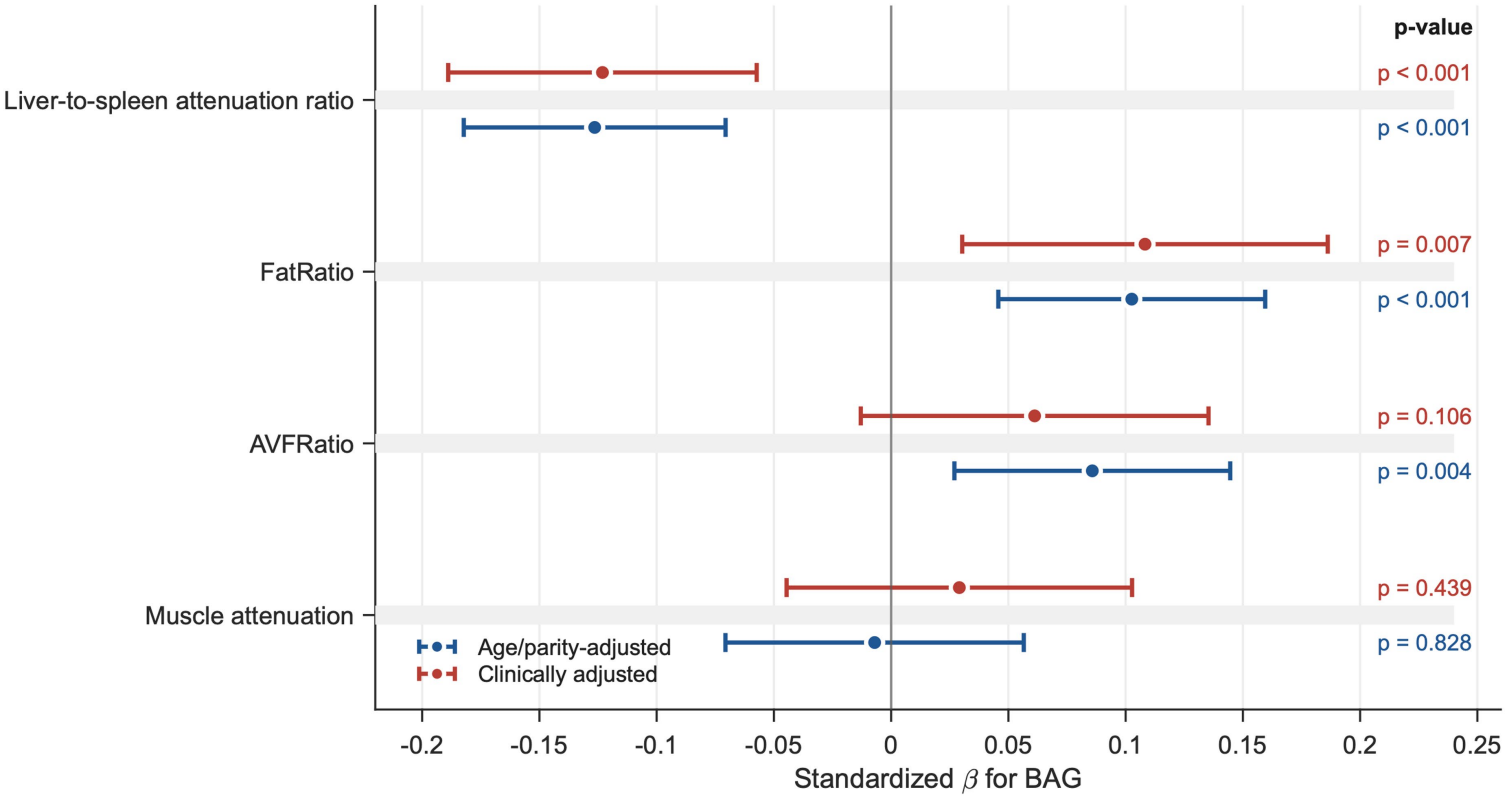
Selected CT-derived metabolic phenotypes associated with the brain age gap. Standardized regression coefficients are shown for the associations between CT-derived phenotypes and BAG. Blue estimates are adjusted for age and parity category. Red estimates were additionally adjusted for *BMI*, hemoglobin *A1c*, triglycerides, *HDL* cholesterol, smoking, and alcohol use. Higher FatRatio and lower liver-to-spleen attenuation ratio were associated with a higher BAG in clinically adjusted models. AVFRatio was defined as abdominal visceral fat area divided by total abdominal fat area, calculated as abdominal visceral fat area/(abdominal visceral fat area + subcutaneous fat area).

Associations between CT-derived phenotypes and BAG are summarized in Table 2 and Figure 2. In the age/parity-adjusted models, a higher FatRatio was associated with a larger BAG (*β* = 0.103, 95% CI 0.046–0.160, *p* < 0.001), and a higher liver-to-spleen attenuation ratio was associated with a lower BAG (*β* = −0.126, 95% CI -0.182 to −0.071, *p* < 0.001). Among the secondary CT markers, AVFRatio and visceral fat volume were also associated with BAG in the age/parity-adjusted models, whereas muscle attenuation was not.

**Table 2:** Associations of CT-derived metabolic phenotypes with standardized brain age gap.

CT-derived phenotype	Model 1				Model 2				Partial *R*^2^
	*n*	*β*	95% CI	*p*-value	*n*	*β*	95% CI	*p*-value	
FatRatio	1,229	0.103	0.046 to 0.160	<0.001***	1,011	0.108	0.030 to 0.186	0.007**	0.0074
Liver-to-spleen attenuation ratio	1,224	−0.126	−0.182 to −0.071	<0.001***	1,006	−0.123	−0.189 to −0.057	<0.001***	0.0134
AVFRatio	1,229	0.086	0.027 to 0.145	0.004**	1,011	0.061	−0.013 to 0.135	0.106	0.0026
Visceral fat volume	1,229	0.073	0.016 to 0.131	0.013*	1,011	0.062	−0.023 to 0.147	0.153	0.0020
Muscle attenuation	1,229	−0.007	−0.071 to 0.057	0.828	1,011	0.029	−0.045 to 0.103	0.439	0.0006
Muscle-to-visceral fat ratio	1,144	−0.093	−0.154 to −0.032	0.003**	933	−0.097	−0.177 to −0.017	0.018*	0.0061

AVF, abdominal visceral fat. Model 1 was adjusted for chronological age and parity category. Model 2 was adjusted for chronological age, parity category, BMI, hemoglobin A1c, triglycerides, HDL cholesterol, smoking status, and alcohol use. Continuous CT predictors and BAG were standardized before regression analyses.

Muscle-to-visceral fat ratio was calculated as single-slice abdominal muscle volume divided by single-slice abdominal visceral fat volume and was evaluated as an exploratory secondary derived marker. Partial *R*^2^ is reported for Model 2.

AVFRatio was defined as the abdominal visceral fat area divided by the total abdominal fat area, calculated as the abdominal visceral fat area/(abdominal visceral fat area + subcutaneous fat area).

Bolded statistical significance is indicated as *p < 0.05, **p < 0.01, and ***p < 0.001.

After adjustment for conventional clinical covariates, including BMI, hemoglobin A1c, triglycerides, HDL cholesterol, smoking, and alcohol use, the primary associations remained significant. A higher FatRatio was associated with a larger BAG (*β* = 0.108, 95% CI 0.030–0.186, *p* = 0.007), and a higher liver-to-spleen attenuation ratio was associated with a lower BAG (*β* = −0.123, 95% CI -0.189 to −0.057, *p* < 0.001). By contrast, the association of AVFRatio was attenuated after clinical adjustment (*β* = 0.061, 95% CI -0.013 to 0.135, *p* = 0.106). Muscle-to-visceral fat ratio remained associated with BAG as an exploratory secondary derived marker (*β* = −0.097, 95% CI -0.177 to −0.017, *p* = 0.018). In the secondary abdominal fat compartment decomposition analyses, log-transformed visceral fat volume, subcutaneous fat volume, and total abdominal fat volume were not independently associated with BAG after clinical adjustment (Supplementary Table S3). By contrast, FatRatio retained its association with larger BAG, whereas AVFRatio was attenuated after clinical adjustment. VIFs for the CT predictors in the corresponding clinically adjusted single-predictor models were as follows: FatRatio, 1.72; liver-to-spleen attenuation ratio, 1.08; and muscle attenuation, 1.44. In a joint abdominal fat and skeletal muscle compartment model, visceral fat volume, subcutaneous fat volume, and skeletal muscle volume were not independently associated with BAG; the corresponding CT compartment VIFs were 2.99, 3.99, and 2.02, respectively.

In exploratory adipose tissue attenuation analyses, higher subcutaneous fat attenuation was associated with higher BAG levels in the clinically adjusted model (*β* = 0.099, 95% CI 0.037–0.161, *p* = 0.002). Higher visceral fat attenuation was also associated with a larger BAG (β = 0.075, 95% CI 0.010–0.140, *p* = 0.023). These associations remained significant after additional adjustment for the corresponding compartment volume (subcutaneous fat attenuation adjusted for subcutaneous fat volume: β = 0.114, *p* < 0.001; visceral fat attenuation adjusted for visceral fat volume: β = 0.113, p = 0.002) and after further adjustment for FatRatio and liver-to-spleen attenuation ratio (subcutaneous fat attenuation: β = 0.093, *p* = 0.005; visceral fat attenuation: β = 0.100, p = 0.005). Subcutaneous fat attenuation and visceral fat attenuation were strongly correlated (r = 0.876). Detailed results are provided in Supplementary Table S4.

Clinically adjusted models for the primary CT phenotypes included 1,006–1,011 complete cases, whereas the muscle-to-visceral fat ratio model included 933 complete cases. The reduction from the full analysis cohort was driven mainly by missing smoking, alcohol, and parity information required for complete-case clinical adjustment rather than by missing CT-derived phenotypes.

In the joint clinical CT model, FatRatio was positively associated with BAG (*β* = 0.095, 95% CI 0.006–0.184, *p* = 0.037), whereas the liver-to-spleen attenuation ratio was inversely associated with BAG (*β* = −0.115, 95% CI -0.184 to −0.047, *p* = 0.001). Muscle attenuation was not independently associated with BAG in the joint clinical model.

### Clinical interpretability and incremental value

In the clinical model, a FatRatio 1 SD higher was associated with 0.78 higher BAG years, and a liver-to-spleen attenuation ratio 1 SD higher was associated with 0.89 lower BAG years. In the joint clinical CT model, a FatRatio of 1 SD higher was associated with 0.68 higher BAG years, and a liver-to-spleen attenuation ratio of 1 SD higher was associated with 0.83 lower BAG years.

**Figure 3:**
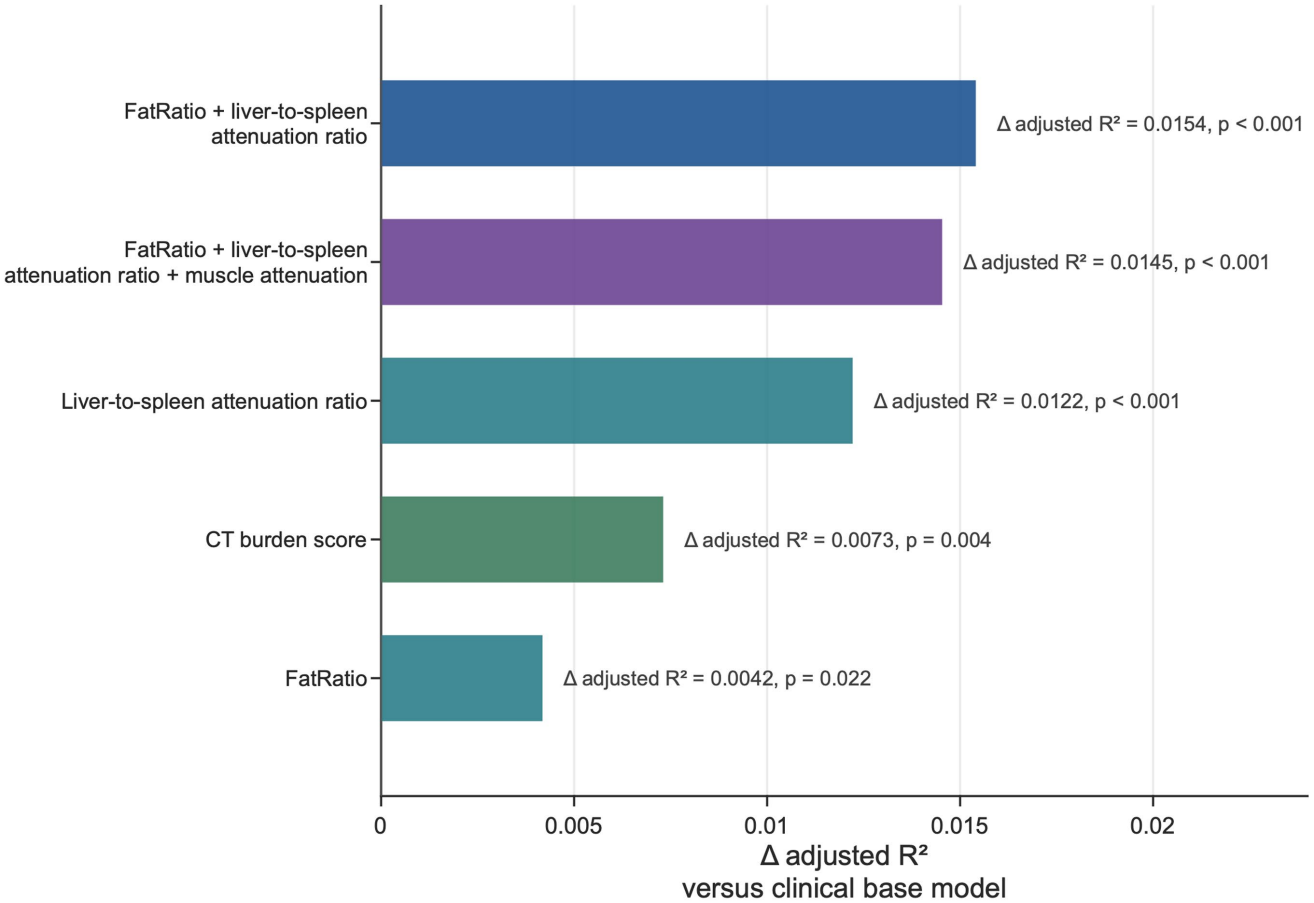
Incremental explanatory value of CT-derived phenotypes. *Δ* adjusted *R*^2^ values are shown for CT-augmented models compared with the clinical base model. The clinical base model included age, parity category, BMI, hemoglobin A1c, triglycerides, HDL cholesterol, smoking, and alcohol use. This figure should be interpreted as an explanatory model comparison rather than evidence of clinical prediction performance.

Incremental explanatory value analyses are summarized in Table 3 and Figure 3. The base clinical model had an adjusted *R*^2^ value of 0.0165. Adding FatRatio increased the adjusted *R*^2^ by 0.0042, whereas adding the liver-to-spleen attenuation ratio increased the adjusted *R*^2^ by 0.0122. Adding both FatRatio and liver-to-spleen attenuation ratio increased the adjusted *R*^2^ by 0.0154, with a partial *R*^2^ of 0.0176 and a nested model *p* < 0.001. Adding FatRatio, liver-to-spleen attenuation ratio, and muscle attenuation yielded a similar partial *R*^2^ value of 0.0178.

**Table 3:** Incremental explanatory value of CT-derived phenotypes beyond clinical covariates.

Model	Adjusted *R*^2^	Δ Adjusted *R*^2^	Partial *R*^2^	Nested *p*-value
Base clinical model	0.0165	–	–	–
Base + FatRatio	0.0207	0.0042	0.0053	0.022*
Base + AVFRatio	0.0183	0.0018	0.0029	0.092
Base + liver-to-spleen attenuation ratio	0.0287	0.0122	0.0134	< 0.001***
Base + integrated CT burden score	0.0238	0.0073	0.0084	0.004**
Base + FatRatio + liver-to-spleen attenuation ratio	0.0319	0.0154	0.0176	< 0.001***
Base + FatRatio + liver-to-spleen attenuation ratio + muscle attenuation	0.0310	0.0145	0.0178	< 0.001***

The base clinical model included age, parity category, BMI, hemoglobin A1c, triglycerides, HDL cholesterol, smoking status, and alcohol use. Δ adjusted *R*^2^ indicates the increase in adjusted *R*^2^ compared with the base clinical model. Partial *R*^2^ and nested p-values were calculated relative to the baseline clinical model. The integrated CT burden score was included in the interpretability analysis and was constructed from a high FatRatio, low liver-to-spleen attenuation ratio, and low muscle attenuation.

AVFRatio was included as a secondary visceral fat distribution phenotype.

Bolded statistical significance is indicated as *p < 0.05, **p < 0.01, and ***p < 0.001.

The integrated CT burden score was evaluated as an exploratory interpretability analysis and is summarized in Supplementary Figure S2 and Supplementary Table S2. In the clinically adjusted ordinal model, a higher CT burden score was associated with a higher standardized BAG level (*β* = 0.131, 95% CI 0.043–0.220, *p* = 0.004). Secondary categorical contrasts are reported in Supplementary Table S2.

### Robustness analyses

In models using quadratic age-bias-corrected BAG, FatRatio remained positively associated with corrected BAG (*β* = 0.122, 95% CI 0.044–0.200, *p* = 0.002), and the liver-to-spleen attenuation ratio remained inversely associated with BAG (*β* = −0.131, 95% CI -0.196 to −0.065, *p* < 0.001). In the joint corrected-BAG model, both FatRatio and the liver-to-spleen attenuation ratio retained independent associations.

After excluding extreme BAG outliers, defined as an absolute BAG z-score greater than 3, FatRatio remained associated with BAG in the clinical model (*β* = 0.101, *p* = 0.009), and the liver-to-spleen attenuation ratio remained inversely associated with BAG (*β* = −0.125, *p* < 0.001). These robustness analyses are summarized in Supplementary Table S1. Odds ratios and apparent AUC values from exploratory high-BAG analyses are summarized in Supplementary Table S2.

Abdominal CT and brain MRI examination dates were available for all 1,280 participants. Abdominal CT and MRI were performed on the same day in 1,097 participants (85.7%), within 30 days in an additional 45 participants (3.5%), and more than 30 days apart in 138 participants (10.8%); the median absolute CT-MRI interval was 0 days. The primary CT-BAG associations remained unchanged after additional adjustment for the CT-MRI interval and showed the same direction in same-day-only sensitivity analyses (Supplementary Results).

In an additional sensitivity analysis addressing glucose-metabolism covariate selection, the primary clinically adjusted models were repeated after replacing hemoglobin A1c with fasting glucose. The primary CT-BAG associations were materially unchanged. FatRatio remained associated with a larger BAG (*β* = 0.109, 95% CI 0.031–0.187, *p* = 0.006), and the liver-to-spleen attenuation ratio remained inversely associated with BAG (*β* = −0.135, 95% CI -0.200 to −0.070, *p* < 0.001).

### Contextual parity analyses

The parity category was not directly associated with mean BAG. Direct parity-BAG associations were not significant in either the full parity-available sample (*p* = 0.545) or the parous-only sample (*p* = 0.603).

The age-by-parity interaction model for BAG adjusted for AVFRatio was significant in the full sample (*p* = 0.023) and only in parous women (*p* = 0.030). Age-restricted sensitivity analyses and model-estimated age-related patterns are summarized in Supplementary Table S1 and Supplementary Figure S3.

## Discussion

In this retrospective health-screening cohort of women with both abdominal CT and brain MRI, CT-derived metabolic phenotypes were associated with the brain age gap. A lower liver-to-spleen attenuation ratio and higher FatRatio were associated with a larger BAG after adjustment for conventional clinical variables, and both retained independent associations in the joint CT models. These associations persisted after age-bias correction of BAG and exclusion of extreme BAG outliers. CT-derived markers added a modest but statistically significant explanatory value beyond BMI and metabolic laboratory covariates. Parity showed no direct association with mean BAG, placing the reproductive-history findings in a contextual rather than a primary explanatory role. Together, these findings position abdominal CT-derived tissue phenotypes as systemic metabolic imaging markers associated with interindividual variation in brain aging, rather than as stand-alone predictors of brain aging at the individual level.

The liver-to-spleen attenuation ratio-based hepatic attenuation phenotype provided the strongest individual CT signal. A lower liver-to-spleen attenuation ratio, derived from dual-energy CT virtual non-contrast images, was associated with larger BAG across primary and sensitivity analyses. This finding is biologically plausible because hepatic steatosis and related metabolic dysfunction have been linked to insulin resistance, inflammation, vascular dysfunction, and liver-brain signaling pathways (Eslam et al., 2020; Mikkelsen et al., 2025). Insulin resistance may also link peripheral metabolic dysfunction to brain function and cognition, providing a broader mechanistic context for metabolic-brain aging associations (Kullmann et al., 2016). Previous studies have linked MASLD, subclinical liver traits, and fatty liver disease with brain age, structural brain markers, and cognitive outcomes (George et al., 2022; Yilmaz et al., 2023; Wang et al., 2025). Our findings extend this literature by showing that a lower liver-to-spleen attenuation ratio, a CT-derived hepatic attenuation phenotype obtainable from routine abdominal imaging, is associated with higher BAG levels, even after adjustment for BMI and metabolic laboratory markers. This supports the relevance of liver-related tissue attenuation in body–brain aging research. At the same time, the liver-to-spleen attenuation ratio should not be interpreted as a formal MASLD diagnosis because MASLD classification requires broader clinical-metabolic assessment, including adequate consideration of alcohol intake and alternative liver disease etiologies.

FatRatio provided complementary body-composition information. Unlike AVFRatio, which reflects the proportion of visceral fat within the total abdominal fat area, FatRatio represents the proportion of abdominal fat area within the fat- and muscle-containing abdominal body-composition area. Thus, FatRatio captures a fat-predominant abdominal body-composition phenotype rather than visceral adiposity alone. In this cohort, AVFRatio was associated with BAG in the age/parity-adjusted models, but this association was attenuated after clinical adjustment. This attenuation should not be interpreted as evidence against the relevance of visceral fat distribution to brain aging. Rather, it may indicate that the association between visceral fat predominance and BAG is partly shared with conventional metabolic factors, including adiposity, glycemic status, lipid profiles, and insulin-resistance-related metabolic pathways. This interpretation is consistent with the close relationship between visceral fat accumulation, insulin resistance, systemic inflammation, and vascular-metabolic dysfunction. By contrast, FatRatio remained associated with BAG after clinical adjustment and in the joint CT models, including liver-to-spleen attenuation ratio, suggesting that relative fat predominance over muscle-containing abdominal tissue may capture additional information beyond conventional metabolic covariates. The abdominal fat compartment decomposition analyses further clarified the interpretation of ratio-based CT phenotypes. Separate visceral fat volume, subcutaneous fat volume, and total abdominal fat volume measures were not independently associated with BAG after clinical adjustment, whereas FatRatio retained its association. This pattern suggests that FatRatio may capture relative abdominal fat predominance over muscle-containing tissue compartments rather than isolated visceral or subcutaneous adiposity alone. However, because ratio-based variables can be influenced by the mathematical coupling between their components, this interpretation should be considered secondary and requires external validation.

The exploratory adipose tissue attenuation analyses further suggest that CT-derived adipose tissue quality may provide information beyond adipose tissue quantity. Prior oncologic CT studies have suggested that adipose tissue attenuation, particularly subcutaneous fat attenuation, may provide prognostic information beyond compartment size and reflect tissue-level metabolic or inflammatory processes (da Cunha et al., 2021; Machado et al., 2024). In the present cohort, higher subcutaneous and visceral fat attenuation values were associated with higher BAG levels after clinical adjustment, and these associations persisted after adjustment for the corresponding compartment volume. However, subcutaneous fat attenuation and visceral fat attenuation were strongly correlated, and therefore, the present findings should be interpreted as evidence of a shared adipose tissue attenuation signal rather than as clearly separable subcutaneous- or visceral-compartment-specific effects. Together, these results support a multidimensional interpretation of CT-derived adiposity phenotypes, distinguishing adipose tissue amount, relative fat predominance, and adipose tissue attenuation as complementary but overlapping dimensions of metabolic body composition. The association of the muscle-to-visceral fat ratio as an exploratory secondary derived marker is also consistent with the potential relevance of relative muscle-fat balance, although this ratio should be interpreted cautiously because it was derived from single-slice compartment measures.

Body MRI studies have linked adipose tissue distribution and visceral adiposity with brain age-related phenotypes (Beck et al., 2022; Moran et al., 2024). Population neuroimaging studies have also associated visceral adiposity and obesity with brain volume and white matter microstructure (Debette et al., 2010; Dekkers et al., 2019). CT studies further support scalable quantification of adiposity phenotypes and have linked visceral adiposity and hepatic steatosis with metabolic syndrome and cardiometabolic outcomes (Pickhardt et al., 2012; Pickhardt et al., 2020; Magudia et al., 2021). Thus, CT-visible adiposity may capture variation in the abdominal metabolic phenotype that is not fully represented by BMI or routine laboratory markers, aligning with a broader shift away from BMI-only metabolic risk assessment toward phenotypes that capture fat distribution, adipose tissue quality, and obesity heterogeneity. Recent reviews and position statements emphasize that visceral and ectopic fat depots can carry a disproportionate cardiometabolic risk compared to the total body size alone (Despres, 2012; Neeland et al., 2018; Neeland et al., 2019).

The integrated CT burden score was retained for exploratory interpretability analysis. Because the score combined a high FatRatio, low liver-to-spleen attenuation ratio, and low muscle attenuation using sample-derived thresholds, it should not be interpreted as a primary mechanistic or predictive model. Although a higher score was associated with a larger BAG, its incremental explanatory value was modest and did not replace the model that included the two primary CT phenotypes. The inclusion of muscle attenuation was based on its role as a recognized CT-derived muscle-quality phenotype reflecting lipid infiltration and tissue quality, rather than on an independent association with BAG in the present cohort (Goodpaster et al., 2000; Aubrey et al., 2014; Engelke et al., 2018; Boutin and Lenchik, 2020). Therefore, the CT burden score should be interpreted as a compact exploratory summary of adverse CT-derived features, and external validation is required.

The incremental value analyses help calibrate the translational interpretation of these findings. Adding FatRatio and liver-to-spleen attenuation ratio to the clinical model improved the explanatory value beyond conventional covariates, but the magnitude of improvement was modest. Thus, these results should not be interpreted as evidence that abdominal CT-derived markers are ready for individual-level brain-aging prediction. Rather, they support a more cautious interpretation: abdominal CT-derived tissue phenotypes may provide additional systemic metabolic information for studying body–brain associations during aging at the population level. This framing aligns with the broader opportunistic CT literature, which emphasizes the extraction of additional health information from routine CT examinations (Pickhardt et al., 2021b; Pickhardt, 2022). Automated CT biomarkers and large-scale body-composition methods provide a technical basis for this approach (Magudia et al., 2021; Pickhardt et al., 2021a). Outcome-oriented studies further highlight the potential of CT-derived biomarkers for risk stratification while also underscoring the need for external validation before clinical deployment (Boutin and Lenchik, 2020; Pickhardt et al., 2020; Pickhardt et al., 2023).

The brain-age phenotype also requires careful interpretation. Although *brainageR* provides a reproducible pretrained framework for estimating brain-predicted age from raw T1-weighted MRI using SPM12-based preprocessing and Gaussian process regression, BAG remains a model-derived imaging phenotype, not a direct measurement of biological aging. In this cohort, the mean raw BAG was −10.29 years, indicating systematic underprediction of chronological age rather than biologically younger brains. This offset may reflect calibration differences when applying a public brain-age model to an independent cohort of Korean women in later adulthood, including differences in age distribution, ethnicity, sex composition, scanner/protocol characteristics, and preprocessing environment. Regression-based brain-age models are also known to show age-related prediction bias, particularly underestimation in older individuals (Beheshti et al., 2019; Smith et al., 2019; de Lange and Cole, 2020; de Lange et al., 2022; Dorfel et al., 2023). Therefore, absolute BAG levels should be interpreted cautiously. The primary inference of this study is based on within-cohort associations between CT-derived phenotypes and BAG, which remained robust after age-bias correction and outlier exclusion.

Parity findings should be interpreted cautiously and contextually. The baseline differences across parity categories should not be overinterpreted because parity groups differed substantially in chronological age. Although higher parity categories showed less favorable metabolic and CT-derived body composition profiles, direct parity-BAG associations were null, indicating that the current data do not support a claim that childbirth directly accelerates brain aging. Pregnancy and childbirth have been associated with long-term changes in body composition and metabolic risk, which may influence later-life metabolic imaging phenotypes (Kim et al., 2016; Schindler et al., 2022). Prior studies relating parity to cognition, dementia, and brain age have reported heterogeneous findings rather than a uniform association (Bae et al., 2020; Ning et al., 2020). In this study, age-by-parity interaction analyses suggested possible contextual patterning; however, these findings were secondary and should not be overinterpreted. Therefore, parity-related analyses were retained as exploratory contextual analyses to account for reproductive history in this women-only cohort transparently, but they do not alter the primary interpretation centered on CT-derived liver attenuation and relative abdominal adiposity.

This study has several strengths. It included a female health-screening cohort with both abdominal CT and brain MRI, which used tissue-level abdominal metabolic phenotypes to examine their relation to a brain MRI-derived aging phenotype. CT phenotyping was performed using dual-energy CT virtual non-contrast images and a deep learning-based body composition and organ attenuation application. The analysis evaluated primary CT associations, joint CT models, BAG-year effect sizes, incremental explanatory value beyond clinical covariates, age-bias-corrected BAG, and outlier sensitivity analyses. This design allowed the study to separate primary CT-BAG associations from secondary or exploratory analyses, including CT burden scores, high-BAG models, and parity-related analyses.

Several limitations should be noted. First, the cross-sectional design prevents causal inference and cannot determine whether adverse CT-derived metabolic phenotypes precede, follow, or co-develop with the older-appearing brain phenotype. Second, the cohort was derived from participants in a single-institution health-screening program who had completed a package including abdominal CT and brain MRI, which may limit the generalizability to broader clinical or community populations. Third, although *brainageR* is a widely used public brain-age tool, the present cohort showed systematic underprediction of chronological age. Therefore, absolute BAG values should be interpreted cautiously, and future studies should validate these findings using population-calibrated brain-age models. Fourth, the liver-to-spleen attenuation ratio is an imaging phenotype rather than a diagnostic MASLD endpoint. The formal MASLD/metabolic dysfunction–associated steatotic liver disease with increased alcohol intake (MetALD) classification was not performed in this study because the study focused on CT-derived hepatic attenuation and body-composition phenotypes rather than adjudicated liver disease categories. Fifth, the CT burden score was sample-derived and requires external validation. Finally, reproductive history was limited, including menopause status, hormone therapy, pregnancy-related complications, breastfeeding history, physical activity, and socioeconomic factors.

In conclusion, abdominal CT-derived metabolic phenotypes reflecting liver attenuation and relative abdominal adiposity were associated with an MRI-derived brain age gap in women. Lower liver-to-spleen attenuation ratio showed the strongest clinically adjusted association, while FatRatio provided complementary adiposity information. These associations persisted after BAG age-bias correction and were not driven by extreme BAG outliers. CT-derived markers added modest but statistically significant explanatory value beyond BMI and metabolic laboratory covariates. Longitudinal and externally validated studies are required before causal or clinical screening claims can be made.

## Data Availability

The data analyzed in this study is subject to the following licenses/restrictions: the dataset used in this study is not publicly available because it contains de-identified but potentially sensitive health-screening data subject to institutional and ethical restrictions. Data may be available from the corresponding author upon reasonable request and with approval from the Institutional Review Board of Seoul National University Hospital. Requests to access these datasets should be directed to ent1127@snu.ac.kr.
