# Supplementary material for: Abdominal CT-derived metabolic phenotypes are associated with the MRI-derived brain age gap in women
Source: Front Aging Neurosci. 2026 Aug 4;18:1893253. doi: 10.3389/fnagi.2026.1893253 (PMC13481376; doi:10.3389/fnagi.2026.1893253)
Supplement: Supplementary file 1 [file Data_Sheet_1.docx]

**Abdominal CT-derived metabolic phenotypes are associated with MRI-derived brain age gap in women**

Chongwon Pae^1,2^, Minchul Kim^3^, Inpyeong Hwang^1,4^, Joon Ho Moon^5,6^, Kyu Sung Choi^1,4,7*^

^1^Department of Radiology, Seoul National University Hospital, Seoul, South Korea

^2^Brain Imaging Center, Seoul National University, Seoul, South Korea

^3^Department of Radiology, Kangbuk Samsung Hospital, Sungkyunkwan University School of Medicine, Seoul, South Korea

^4^Department of Radiology, Seoul National University College of Medicine, Seoul, South Korea

^5^Department of Endocrinology and Metabolism, Seoul National University Bundang Hospital, Seongnam, South Korea

^6^Department of Internal Medicine, Seoul National University College of Medicine, Seoul, South Korea

^7^Healthcare AI Research Institute, Seoul National University Hospital, Seoul, South Korea

**Supplementary Materials**

Supplementary Results

Supplementary Tables S1-S4

Supplementary Figures S1-S3

**Supplementary Results**

In exploratory logistic models defining high BAG as the top quartile of BAG, liver-to-spleen attenuation ratio was inversely associated with high BAG (OR = 0.841 per 1 SD higher, 95% CI 0.723 to 0.978, *p* = 0.025), and CT burden score was positively associated with high BAG (OR = 1.252 per 1-point increase, 95% CI 1.023 to 1.532, *p* = 0.029). FatRatio showed a positive but not statistically significant association with high BAG (OR = 1.196, 95% CI 0.982 to 1.456, *p* = 0.075). Apparent AUC increased modestly from 0.591 for the clinical base model to 0.608 after adding FatRatio, liver-to-spleen attenuation ratio, and muscle attenuation.

**CT-MRI temporal interval sensitivity analyses**

Examination dates for abdominal CT and brain MRI were available for all 1,280 participants. CT and MRI were performed on the same day in 1,097 participants (85.7%) and within 30 days in 1,142 participants (89.2%). The median absolute CT-MRI interval was 0 days. To evaluate whether variability in the CT-MRI interval influenced the primary findings, the clinically adjusted models were repeated with additional adjustment for the absolute CT-MRI interval. The primary associations were essentially unchanged. FatRatio remained associated with larger BAG (*β* = 0.108, *p* = 0.007), and liver-to-spleen attenuation ratio remained inversely associated with BAG (*β* = -0.124, *p* < 0.001). The CT-MRI interval itself was not associated with BAG (*β* = 0.011, 95% CI -0.048 to 0.070, *p* = 0.712). In same-day-only analyses, the associations showed the same direction for FatRatio (*n* = 874, *β* = 0.105, *p* = 0.011) and liver-to-spleen attenuation ratio (*n* = 870, *β* = -0.170, *p* < 0.001). These findings indicate that the primary CT-BAG associations were not driven by variability in the temporal interval between abdominal CT and brain MRI.

**Supplementary Tables**

**Supplementary Table S1. BAG calibration, robustness, contextual parity analyses**

| **Analysis domain** | **Analysis or predictor** | **Sample/model** | **Estimate** | **95% CI** | **p value** |
| --- | --- | --- | --- | --- | --- |
| **BAG calibration** | Mean predicted brain age, years | Parity-available analytic cohort | 50.93 | - | - |
| **BAG calibration** | Mean chronological age, years | Parity-available analytic cohort | 61.22 | - | - |
| **BAG calibration** | Mean BAG, years | Parity-available analytic cohort | -10.29 ± 7.22 | - | - |
| **BAG calibration** | Correlation between BAG and chronological age | Parity-available analytic cohort | r = 0.040 | - | 0.157 |
| **Age-bias corrected BAG** | FatRatio | Clinical model | β = 0.122 | 0.044 to 0.200 | **0.002**** |
| **Age-bias corrected BAG** | Liver-to-spleen attenuation ratio | Clinical model | β = -0.131 | -0.196 to -0.065 | **< 0.001***** |
| **Outlier-excluded BAG** | FatRatio | Absolute BAG z-score ≤3 | β = 0.101 | - | **0.009**** |
| **Outlier-excluded BAG** | Liver-to-spleen attenuation ratio | Absolute BAG z-score ≤3 | β = -0.125 | - | **< 0.001***** |
| **Contextual parity analysis** | Direct parity-BAG association | Full parity-available sample | - | - | 0.545 |
| **Contextual parity analysis** | Direct parity-BAG association | Parous-only sample | - | - | 0.603 |
| **Contextual parity analysis** | Age × parity interaction for BAG adjusted for AVFRatio | Full parity-available sample | - | - | **0.023*** |
| **Contextual parity analysis** | Age × parity interaction for BAG adjusted for AVFRatio | Parous-only sample | - | - | **0.030*** |
| **Contextual parity analysis** | Age × parity interaction for BAG adjusted for AVFRatio | Women aged ≥55 years | - | - | **0.016*** |
| **Contextual parity analysis** | Age × parity interaction for BAG adjusted for AVFRatio | Parous women aged ≥55 years | - | - | **0.007**** |

Note: BAG, brain age gap; AVF, abdominal visceral fat

Statistical significance is indicated as *p < 0.05, **p < 0.01, ***p < 0.001.

**Supplementary Table S2. CT burden score and exploratory high-BAG analyses**

| **Analysis domain** | **Analysis or predictor** | **Model** | **n** | **Effect estimate** | **95% CI** | **p value** | **AUC** |
| --- | --- | --- | --- | --- | --- | --- | --- |
| **CT burden score** | Per 1-point increase | Age/parity-adjusted model | 1224 | 0.152 | 0.082 to 0.222 | **<0.001***** | - |
|  | Per 1-point increase | Clinically adjusted model | 1006 | 0.131 | 0.043 to 0.220 | **0.004**** | - |
|  | CT burden score ≥2 versus 0 | Clinically adjusted model | 656 (score 0 n = 461; score ≥2 n = 195) | 0.258 | 0.048 to 0.468 | **0.016*** | - |
| **High-BAG exploratory model** | Base clinical model | Clinical logistic model | - | - | - | - | 0.591 |
|  | FatRatio, per 1 SD higher | Clinical logistic model | - | OR = 1.196 | 0.982 to 1.456 | 0.075 | - |
|  | Liver-to-spleen attenuation ratio, per 1 SD higher | Clinical logistic model | - | OR = 0.841 | 0.723 to 0.978 | **0.025*** | - |
|  | CT burden score, per 1-point increase | Clinical logistic model | - | OR = 1.252 | 1.023 to 1.532 | **0.029*** | - |
|  | Clinical model + FatRatio + liver-to-spleen attenuation ratio + muscle attenuation | Clinical logistic model | - | - | - | - | 0.608 |

Note: BAG, brain age gap; SD, stanard deviation; AUC, area under curve

Model-specific n varied according to complete-case availability.

Statistical significance is indicated as *p < 0.05, **p < 0.01, ***p < 0.001.

**Supplementary Table S3. Abdominal fat and muscle compartment decomposition analyses**

| **Analysis** | **CT-derived phenotype** | **Model** | **Standardized β**  **(95% CI)** | **p value** | **Partial R²** | **CT predictor VIF** |
| --- | --- | --- | --- | --- | --- | --- |
| **Primary ratio phenotype** | **FatRatio** | **Clinically adjusted** | **0.108**  **(0.030 to 0.186)** | **0.007*** | **0.0074** | **1.72** |
| Visceral proportion phenotype | AVFRatio | Clinically adjusted | 0.061  (-0.013 to 0.135) | 0.106 | 0.0026 | 1.48 |
| Fat compartment volumes | Visceral fat volume | Clinically adjusted | 0.062  (-0.023 to 0.147) | 0.153 | 0.0020 | 2.01 |
| Fat compartment volumes | Subcutaneous fat volume | Clinically adjusted | -0.003  (-0.100 to 0.095) | 0.959 | 0.0000 | 2.69 |
| Fat compartment volumes | Total abdominal fat volume | Clinically adjusted | 0.025  (-0.074 to 0.125) | 0.618 | 0.0003 | 2.79 |
| Muscle phenotypes | Skeletal muscle volume | Clinically adjusted | -0.048  (-0.129 to 0.034) | 0.253 | 0.0013 | 1.86 |
| Muscle phenotypes | Muscle attenuation | Clinically adjusted | 0.029  (-0.045 to 0.103) | 0.439 | 0.0006 | 1.44 |
| Joint fat and muscle compartment model | Visceral fat volume | Joint compartment | 0.046  (-0.059 to 0.152) | 0.388 | 0.0007 | 2.99 |
| Joint fat and muscle compartment model | Subcutaneous fat volume | Joint compartment | -0.024  (-0.143 to 0.096) | 0.696 | 0.0002 | 3.99 |
| Joint fat and muscle compartment model | Skeletal muscle volume | Joint compartment | -0.052  (-0.137 to 0.034) | 0.235 | 0.0014 | 2.02 |

Note:

Clinically adjusted models were adjusted for chronological age, parity category, body mass index, hemoglobin A1c, triglycerides, HDL cholesterol, smoking status, and alcohol use.

The joint compartment model included visceral fat volume, subcutaneous fat volume, and skeletal muscle volume simultaneously with the same clinical covariates.

Statistical Significance: *p<0.05

**Supplementary Table S4. Exploratory adipose tissue attenuation analyses**

| **Predictor** | **Model** | **n** | **standardized β** | **95% CI** | **p** | **Partial R^2^** | **VIF** |
| --- | --- | --- | --- | --- | --- | --- | --- |
| SF attenuation | Age/parity-adjusted | 1,229 | 0.093 | 0.038 to 0.149 | **<0.001**** | 0.0088 | 1.00 |
| SF attenuation | Clinically adjusted | 1,011 | 0.099 | 0.037 to 0.161 | **0.002*** | 0.0098 | 1.08 |
| AVF attenuation | Age/parity-adjusted | 1,229 | 0.049 | -0.007 to 0.105 | 0.086 | 0.0024 | 1.01 |
| AVF attenuation | Clinically adjusted | 1,011 | 0.075 | 0.010 to 0.140 | **0.023*** | 0.0052 | 1.20 |
| SF attenuation | Clinically adjusted + SF volume | 1,011 | 0.114 | 0.047 to 0.180 | **<0.001**** | 0.0112 | 1.25 |
| AVF attenuation | Clinically adjusted + AVF volume | 1,011 | 0.113 | 0.042 to 0.183 | **0.002**** | 0.0097 | 1.43 |
| SF attenuation | Clinically adjusted + FatRatio + liver-to-spleen attenuation ratio | 1,006 | 0.093 | 0.028 to 0.159 | **0.005*** | 0.0079 | 1.21 |
| AVF attenuation | Clinically adjusted + FatRatio + liver-to-spleen attenuation ratio | 1,006 | 0.100 | 0.030 to 0.170 | **0.005*** | 0.0078 | 1.42 |
| SF attenuation | SF + AVF attenuation joint model | 1,011 | 0.174 | 0.036 to 0.312 | **0.014*** | 0.0061 | 5.41 |
| AVF attenuation | SF + AVF attenuation joint model | 1,011 | -0.087 | -0.232 to 0.057 | 0.236 | 0.0014 | 5.99 |
| SF attenuation | Full exploratory CT model | 1,006 | 0.051 | -0.099 to 0.200 | 0.506 | 0.0004 | 6.36 |
| AVF attenuation | Full exploratory CT model | 1,006 | 0.051 | -0.109 to 0.212 | 0.532 | 0.0004 | 7.43 |

Notes: SF, Subcutaneous Fat; AVF, Abdominal Visceral Fat;

Age/parity-adjusted models included chronological age and parity category.

Clinically adjusted models included chronological age, parity category, BMI, hemoglobin A1c, triglycerides, HDL cholesterol, smoking status, and alcohol use.

SF and AVF volume-adjusted models additionally adjusted for the corresponding log-transformed adipose tissue volume.

Primary CT-adjusted models additionally adjusted for FatRatio and liver-to-spleen attenuation ratio.

The full exploratory CT model included SF attenuation, AVF attenuation, FatRatio, and liver-to-spleen attenuation ratio simultaneously with clinical covariates.

Statistical significance: *p<0.05, **p<0.005

**Supplementary Figures**

**Supplementary Figure S1.** Brain age calibration and brain age gap (BAG) age-bias diagnostic. Scatterplots showing predicted brain age versus chronological age and BAG, calculated as predicted brain age minus chronological age, versus chronological age. These plots support cohort-level BAG calibration and age-bias diagnostics and are intended for supplementary verification rather than as a main Results claim.

**Supplementary Figure S2.** Integrated CT burden score and brain age gap. Mean standardized BAG according to the exploratory integrated CT burden score. Error bars indicate standard errors. Statistical results are provided in Supplementary Table S2.

**Supplementary Figure S3.** Parity as contextual life-course information for age-related CT and brain age gap (BAG) patterns. Model-estimated age-related patterns according to parity category. Panel A shows age-related patterns in AVFRatio. Panel B shows age-related BAG patterns after adjustment for AVFRatio. These analyses support parity as contextual life-course information for age-related metabolic-brain patterning rather than as a direct determinant of mean BAG.
